# Down-regulation of *RBM47* due to diminished activation by forkhead box A1 (FOXA1) and silencing by CpG methylation is associated with epithelial-mesenchymal transition and metastasis of colorectal cancer

**DOI:** 10.1186/s43556-025-00382-4

**Published:** 2025-12-03

**Authors:** Matjaz Rokavec, Yuyun Du, Heiko Hermeking

**Affiliations:** 1 Experimental and Molecular Pathology, Institute of Pathology, Faculty of Medicine, Ludwig-Maximilians-Universität München, Thalkirchner Strasse 36, Munich, 80337 Germany; 2 German Cancer Consortium (DKTK), Partner Site Munich, Munich, 80336 Germany; 3 German Cancer Research Center (DKFZ), Heidelberg, 69120 Germany

**Keywords:** Colorectal cancer, Metastasis, RBM47, FOXA1, DNA methylation, Mesenchymal- epithelial transition/MET

## Abstract

The gene encoding the RNA-binding motif protein 47 (RBM47) is highly expressed in epithelial cells and its down-regulation is characteristic for many types of cancer, among them colorectal cancer (CRC). However, the underlying mechanisms for this differential expression of *RBM47* and its functional consequences during CRC progression have remained unknown. Here we found that *RBM47* expression progressively decreases during CRC progression and is associated with poor prognosis and the metastatic CRC subtypes CMS4 and CRIS-B. In mice and humans *RBM47* expression was highest in endoderm-derived tissues. The expression of forkhead box A1 (*FOXA1)*, a transcription factor essential for the development of endoderm-derived epithelial tissues, showed a positive correlation with *RBM47* expression in human tissues, as well as in primary CRCs and derived cell lines. Like RBM47, *FOXA1* showed a down-regulation during CRC progression that is associated with poor prognosis and CMS4/CRIS-B. Ectopic FOXA1 induced *RBM47* via directly binding to FOXA1 binding sites within the *RBM47* promoter region. Up-regulation of *RBM47* was necessary for FOXA1-mediated mesenchymal-to-epithelial transition (MET) and inhibition of CRC cell migration and invasion. *RBM47* expression was silenced by CpG methylation in mesenchymal-like CRC cell lines. Moreover, epigenetic silencing of *RBM47* in primary CRCs was associated with liver metastases. Therefore, the down-regulation of *RBM47* is presumably initially mediated by loss of *FOXA1* expression and subsequently fixed by CpG methylation of the *RBM47* promoter. This down-regulation of *RBM47* facilitates EMT and thereby promotes CRC metastasis. Finally, our results show that CpG hypermethylation of the *RBM47* promoter represents a potential biomarker for metastatic CRC.

**Supplementary Information:**

The online version contains supplementary material available at 10.1186/s43556-025-00382-4.

## Introduction

Colorectal cancer (CRC) causes more than 900,000 deaths worldwide every year making it the second most lethal type of cancer [1]. More than 90% of CRC mortality is due to metastasis and less than 15% of patients with metastatic CRC (mCRC) survive more than 5 years [2]. Therefore, a better understanding of mechanisms underlying CRC metastasis is needed to improve the prevention and therapy of mCRC.

The RNA binding motif protein 47 (RBM47) is an RNA-binding protein that regulates the post-transcriptional expression of its targets by modulating the stability, splicing, and editing of RNA [3]. RBM47 acts tumor suppressive in breast, lung and CRC cell lines and mouse models [4–6]. The down-regulation of *RBM47* has been consistently associated with aggressive cancer phenotypes, making it a promising biomarker for CRC progression [3]. RBM47 suppresses breast cancer progression and metastasis by stabilizing the *Dickkopf 1* (*DKK1)* mRNA and thereby inhibiting Wnt activity [6]. Furthermore, RBM47 suppresses lung tumor growth through the inhibition of NFE2-like bZIP transcription factor 2 (NRF2) activity [5]. We have previously shown that inactivation of *RBM47* in a human, epithelial-like CRC cell line allows metastases formation in mice [4]. Recently, intestinal epithelial-specific deletion of *Rbm47* was shown to cause polyposis in aged mice, whereas the same mice were protected against colitis-associated cancer, suggesting that the role of RBM47 in carcinogenesis may depend on the context [7].

A critical aspect of metastasis is the dynamic regulation of epithelial-mesenchymal plasticity, which facilitates tumor dissemination and colonization at distant sites [8]. Accumulating evidence suggests, that RBM47 plays a pivotal role in maintaining epithelial integrity by antagonizing epithelial-to-mesenchymal transition (EMT) [4, 5, 9, 10]. *RBM47* is highly expressed in epithelial-like cancer cells, whereas decreased *RBM47* expression was found in mesenchymal-like cancer cells, which are generally more invasive and metastatic than epithelial-like CRC lines [4]. However, the mechanisms underlying *RBM47* repression during cancer progression are not well understood. DNA methylation is a common mechanism of tumor suppressor gene inactivation [11], raising the possibility that down-regulation of *RBM47* in CRC may be due to epigenetic silencing.

*RBM47* is repressed by several EMT-related transcription factors, such as snail family transcriptional repressor 1 (SNAIL), snail family transcriptional repressor 2 (SLUG), signal transducer and activator of transcription 3 (STAT3), and SMAD family member 3 (SMAD3) in cancer cells [4, 5]. However, it is not known which transcription factors (TFs) or pathways are driving the high expression of *RBM47* observed in normal epithelial tissues. The forkhead box A1 (FOXA1) protein is a member of the FOXA family of fork-head domain TFs, which play essential roles in the development of endoderm and endoderm-derived epithelial tissues [12]. FOXA1 and FOXA2 TFs are suppressors of EMT and metastasis in lung and pancreatic cancer [13, 14]. Interestingly, the EMT transcription factor SNAIL directly suppresses FOXA1 expression in CRC cells, which facilitates the inactivation of FOXA1-bound enhancers at key genes associated with epithelial differentiation, such as E-cadherin (*CDH1)*, caudal type homeobox 2 (*CDX2)*, and EPH receptor B3 (*EPHB3)* [15]. Like *RBM47*, *FOXA1* is highly expressed in normal colon epithelial cells, but significantly down-regulated in colon cancers [16].

In this study, we show that *RBM47* expression is directly induced by FOXA1 and strictly correlates with *FOXA1* expression in normal tissues and during CRC progression. Moreover, the induction of *RBM47* by FOXA1 was required for FOXA1-induced mesenchymal-to-epithelial transition (MET) and the repression of migration and invasion in CRC cells. In addition, *RBM47* was found to be silenced by CpG methylation in mesenchymal-like CRC cell lines and preferentially in primary CRCs from patients with liver metastases.

## Results

### Association of *RBM47* down-regulation in CRCs with metastasis and poor prognosis

**Fig. 1:**
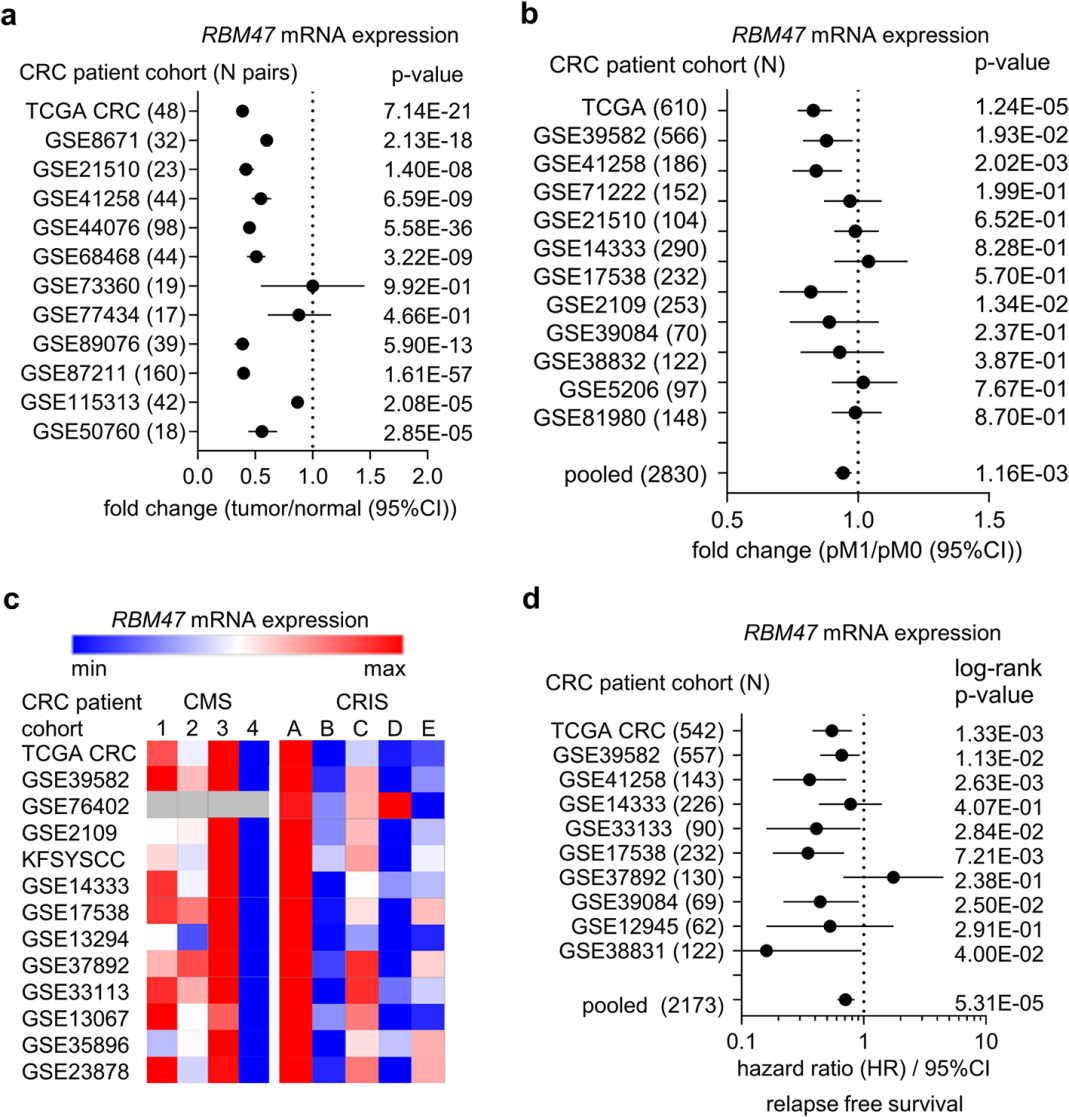
Associations of *RBM47* expression with clinico-pathological characteristics in CRC. **a** *RBM47* expression in colorectal tumors and matched adjacent normal colon tissues in indicated patient cohorts. Fold changes are represented by dots and 95% confidence intervals (CI) by horizontal lines. **b** Forest plot showing fold changes in *RBM47* expression between primary colorectal tumors from patients with metastasis (pM1) and without metastasis (pM0) in indicated patient cohorts. Dots represent fold changes and horizontal lines show 95% (CI). **c** Expression of *RBM47* in human colorectal tumors belonging to indicated CMS and CRIS subtypes. **d** Forest plot showing Hazard ratios for relapse free survival by comparing patients with high versus low expression of *RBM47* in indicated CRC patient cohorts. Hazard ratios are represented by dots and 95% CIs by horizontal lines. The log-rank method was used to calculate *p*-values

We have previously shown that *RBM47* mRNA expression is significantly lower in tumors when compared to adjacent normal colon tissue by analyzing The Cancer Genome Atlas (TCGA) colon cancer patient cohort representing 39 matched tumor/normal tissue pairs [4]. Here these analyses were extended to 12 cohorts representing 584 CRC patients: 10 of these showed consistent and significant down-regulation of *RBM47* in tumors when compared to adjacent normal tissue (Fig. 1a). Furthermore, low *RBM47* expression was consistently associated with distant metastasis (M1; Fig. 1b). A consensus molecular subtype (CMS) classification has been introduced that groups CRCs into 4 main subtypes [17]. The expression of *RBM47* was lowest in the mesenchymal-like CMS4 subtype (Fig. 1c), which corresponds to patients with the poorest prognosis and highest incidence of metastases. CMS subtypes were defined by expression signatures derived from bulk tumor samples, which also contain stromal cells that might confound the results. To circumvent this problem, mRNA expression signatures were obtained from patient-derived xenografts (PDXs) using microarray analyses, in which human-specific probe sets were used to selectively eliminate the contribution of (murine) stromal mRNAs from whole-tumor mRNA expression patterns. Thereby, 5 different colorectal cancer intrinsic subtypes (CRIS) were defined [18]. *RBM47* expression was lowest in the CRIS-B subtype (Fig. 1c), which is also characterized by poor prognosis and metastasis. Finally, low *RBM47* expression was consistently associated with poor relapse-free survival (Fig. 1d) of CRC patients. Taken together, compared to normal colon tissue, *RBM47* expression is consistently down-regulated in tumors. Furthermore, low *RBM47* expression is associated with distant metastasis and poor survival.

### In vivo correlation of RBM47 and FOXA1 expression

**Fig. 2:**
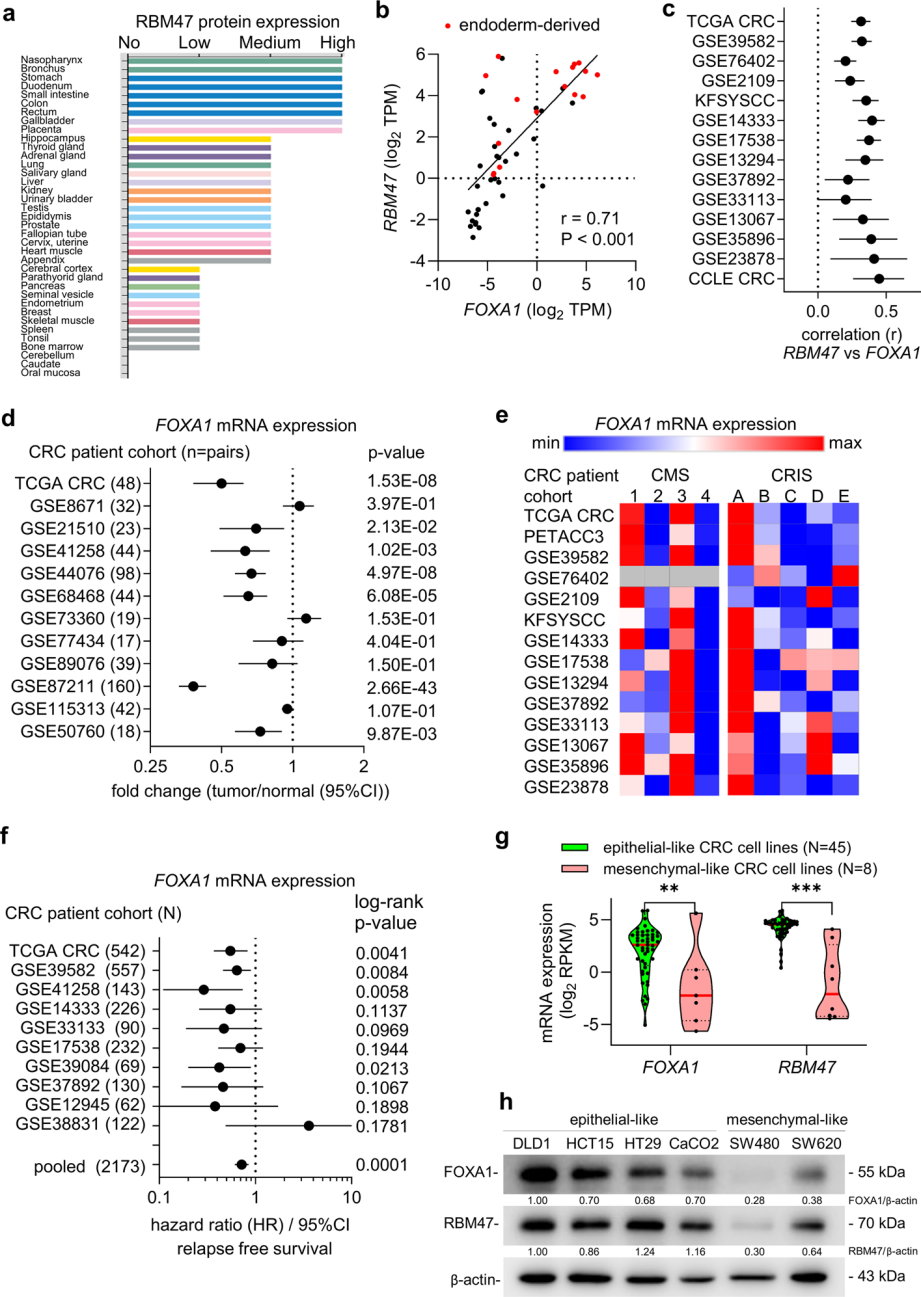
FOXA1 is a regulator of *RBM47* expression. **a** RBM47 protein expression in human tissues (data from the Human Protein Atlas). **b** Correlation between *FOXA1* and *RBM47* expression in adult human tissues (data from GTEx). The Pearson correlation coefficient with two-tailed *p*-value is shown. **c** Correlation between *FOXA1* and *RBM47* mRNA expression in colorectal tumors and cell lines from indicated cohorts. Pearson correlation coefficients (r) and 95% CIs are shown. **d** *FOXA1* expression in colorectal tumors and matched adjacent normal colonic mucosa in indicated patient cohorts. Fold changes are represented by dots and 95% CIs by horizontal lines. **e** Expression of *FOXA1* in human colorectal tumors corresponding to indicated CMS and CRIS subtypes. **f** Relapse free survival of CRC patients with high versus low expression of *FOXA1*. Hazard ratios are represented by dots and 95% CIs by horizontal lines. The log-rank method was used to calculate *p*-values. **g** *FOXA1* and *RBM47* expression in epithelial- and mesenchymal-like CRC cell lines. **h** Western blot analysis of FOXA1 and RBM47 protein expression within a panel of epithelial-like and mesenchymal-like colorectal cancer cell lines. Western blot replicates 2 and 3 are shown in Fig. S2. ** *p* < 0.01; ***, *p* < 0.001

Next, we aimed to determine which transcription factors (TFs) are responsible for the elevated expression of *RBM47* in normal epithelial cells and early-stage CRC. In humans, RBM47 protein expression is highest in the colon, small intestine, gallbladder and lung (Fig. 2a; data from the Human Protein Atlas) tissues, which are derivatives of the endoderm. Indeed, RBM47 is preferentially expressed in the endoderm of E8.5 mouse embryos and in endoderm-derived tissues in adult mice [19]. Therefore, endoderm-specific TFs might bind to the *RBM47* promoter region and activate its transcription. The members of the FOXA family of fork-head domain TFs play essential roles in the development of endoderm and endoderm-derived tissues [12]. Analysis of RNA-Seq data from the Genotype-Tissue Expression project (GTEx) showed a significant positive correlation between *RBM47* and *FOXA1* expression across human tissues with high expression of both mRNAs in endoderm-derived tissues (Fig. 2b). Furthermore, *FOXA1* mRNA expression displayed a consistent and statistically significant positive correlation with *RBM47* mRNA expression in primary CRCs and CRC cell lines in 14 cohorts (Fig. 2c). Like *RBM47*, *FOXA1* expression was consistently and significantly down-regulated in colon cancers when compared to adjacent normal colon tissue (Fig. 2d). Interestingly, the fold changes of *RBM47* and *FOXA1* expression between tumor and normal tissues within the analyzed CRC patient cohorts showed a significant, positive correlation (Fig. S1; data from Figs. 1a and 2d). Furthermore, similar to *RBM47*, *FOXA1* expression was lowest in the CMS4 CRC subtype (Fig. 2e) and low *FOXA1* expression was consistently associated with low relapse-free survival in CRC patients (Fig. 2f). Finally, *FOXA1* and *RBM47* expression was lower in mesenchymal-like CRC cell lines when compared to epithelial-like CRC cell lines (Fig. 2g-h, Fig. S2). In the CRC field the SW480 and SW620 cell lines are considered to represent mesenchymal-like CRC cell lines, whereas DLD-1, HCT15, HT29, and CaCO2 represent epithelial-like CRC cell lines [20]. Here, the epithelial- and mesenchymal-like status of CRC cell lines was defined based on the expression of the epithelial marker E-cadherin (CDH1) and the mesenchymal marker Vimentin (VIM), which we determined previously [21]. Epithelial-like cells lines express high levels of CDH1 and low levels of VIM, whereas mesenchymal-like cell lines express low levels of CDH1 and high levels of VIM. Taken together, these results implied that FOXA1 may directly induce *RBM47* expression.

### *RBM47* expression is directly induced by FOXA1

**Fig. 3:**
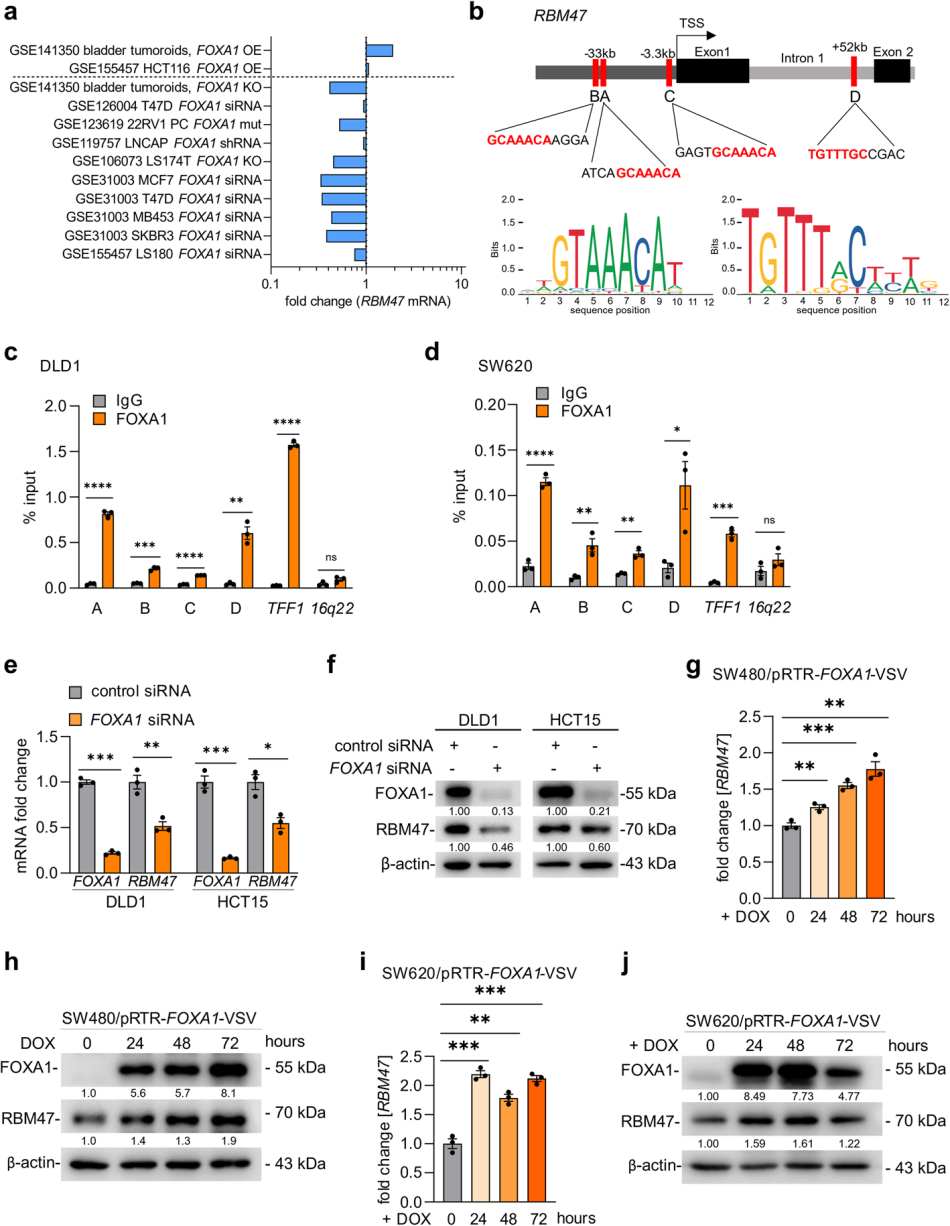
FOXA1 directly induces *RBM47* expression. **a** Meta-analysis of *RBM47* expression in datasets representing studies of ectopic expression or knock-down of FOXA1 in the indicated cell lines. **b** Map of human *RBM47* genomic region with indicated FOXA1 binding sites. **c-d** qChIP analysis of FOXA1 occupancy at the indicated *RBM47* genomic regions in DLD1 **c** and SW620 **d** cells. Chromatin was enriched by anti-FOXA1 or anti-rabbit-IgG antibodies. *TFF1* and *16q22* served as positive and negative controls, respectively. **e–f** qPCR (E) and Western blot (F) analyses of FOXA1 and RBM47 expression in DLD1 and HCT15 cells after 72 h after transfection of *FOXA1*-specific siRNA. **g-j** qPCR and Western blot analyses of FOXA1 and RBM47 expression in SW480/pRTR-*FOXA1-VSV* and SW620/pRTR-*FOXA1-VSV* cells treated with DOX for indicated time points. Mean values ± SD (*n* = 3) are provided. *p < 0.05; **p < 0.01; *** *p* < 0.001

To investigate whether *RBM47* expression is directly regulated by FOXA1, public GEO expression profiling datasets with ectopic expression/knockdown/knockout of *FOXA1* in cell lines or mice were analyzed (Fig. 3a). *RBM47* mRNA was induced after ectopic *FOXA1* expression and suppressed after *FOXA1* knockdown/knockout in the majority of studies (Fig. 3a). Next, three putative FOXA1 binding sites upstream of the transcriptional start site (TSS) and one within the first intron of the *RBM47* locus were determined (Fig. 3b). Besides one binding site 3.3 kbp upstream of the TSS, three putative binding sites were located more than 30 kb from the TSS, suggesting that they are located within enhancer regions. This is consistent with previous reports, which showed that FOXA1 mainly operates through binding to enhancers [22]. Analysis of FOXA1 chromatin immuno-precipitation (ChIP)-seq data sets from various cell lines and tissues (available via the Encyclopedia of DNA Elements (ENCODE) consortium) suggested that FOXA1 binds to the predicted binding sites A and D in the *RBM47* locus (Fig. S3). ChIP-qPCR analysis confirmed the binding of FOXA1 to the predicted FOXA1 binding sites A/B and D and also to the binding site C in DLD1 and SW620 CRC cells (Fig. 3c-d). Next, FOXA1 was suppressed using RNA-interference in DLD1 and HCT15 cells, which express high levels of FOXA1, and ectopically expressed in SW480 and SW620 cells, which display low levels of FOXA1. The repression of FOXA1 by a pool of four different siRNAs resulted in a significant repression of RBM47 mRNA and protein in DLD1 and HCT15 CRC cells (Fig. 3e-f). Furthermore, ectopic expression of FOXA1 in SW480 and SW620 CRC cells resulted in the induction of RBM47 mRNA and protein expression (Fig. 3g-j). Altogether, these results imply that *RBM47* is a direct target of FOXA1.

### FOXA1 induces mesenchymal to epithelial transition (MET) via up-regulation of *RBM47*

**Fig. 4:**
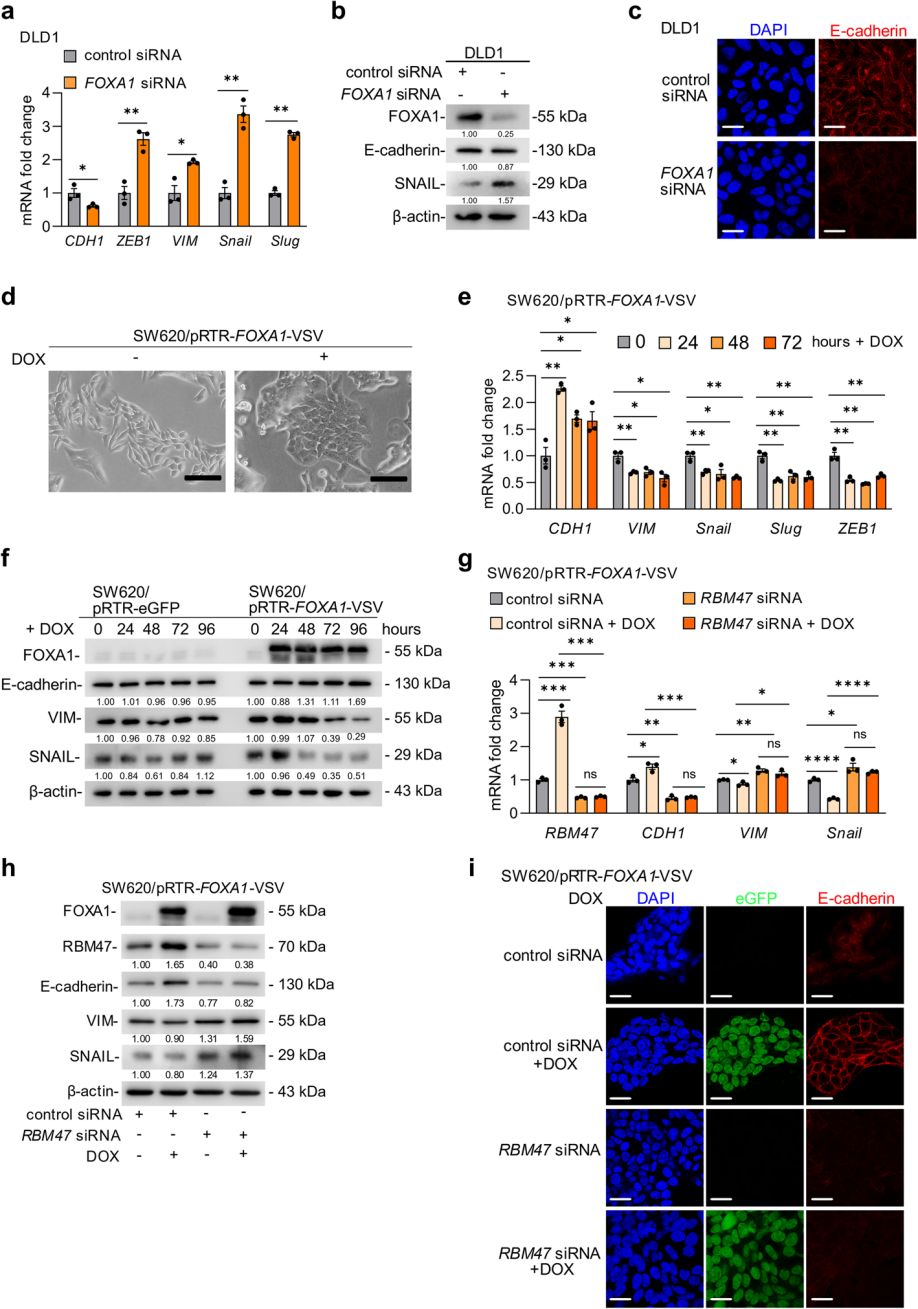
FOXA1 induces MET via inducing *RBM47*. **a** qPCR analyses of indicated mRNAs in DLD1 cells 72 h after *FOXA1* siRNA transfection. **b** Western blot analysis of indicated proteins in DLD1 cells 72 h after *FOXA1* siRNA transfection. **c** Indirect immunofluorescence (IF) analysis of E-cadherin in DLD1 cells 72 h after *FOXA1* siRNA transfection. **d** Representative phase-contrast images of SW620 cells after ectopic FOXA1 expression for 72 h. Scale bars, 50 μm. **e–f** qPCR and Western blot analyses of FOXA1, CDH1, VIM, and SNAIL mRNA and protein expression in SW620 cells transfected with pRTR-eGFP or pRTR-*FOXA1*-VSV vectors, treated with or without doxycycline (DOX) for the indicated periods. **g-h** qPCR and Western blot analyses of MET/EMT markers (CDH1, VIM, SNAIL) after ectopic expression of FOXA1 and/or RBM47 knock-down in SW620 cells. **i** IF analysis of E-cadherin in SW620/pRTR-*FOXA1*-VSV cells treated with vehicle or DOX and control or *RBM47* siRNA. Mean values ± SD (*n* = 3) are provided. *p < 0.05; **p < 0.01; *** *p* < 0.001; **** *p* < 0.0001

Since FOXA1 and RBM47 have been associated with EMT/MET and *RBM47* is a FOXA1 target, we investigated whether FOXA1 regulates EMT/MET via RBM47. The siRNA-mediated knockdown of FOXA1 in epithelial-like DLD1 cells induced the expression of mesenchymal markers *ZEB1, VIM, SNAIL*, and *SLUG* and decreased the expression of the epithelial marker E-cadherin (Fig. 4a-c). Therefore, elevated levels of FOXA1 expression are required to maintain CRC cells in an epithelial-like state. Furthermore, down-regulation of FOXA1 may be sufficient for the induction of EMT. On the other hand, ectopic expression of FOXA1 in the mesenchymal-like CRC cell-lines SW480 and SW620 promoted a transition from a mesenchymal to an epithelial morphology (spindle-shaped cells with a scattered growth pattern became tightly packed, cobble-stone like cells), indicating that FOXA1 induces MET in CRC cells (Fig. 4d, Fig. S4a). Indeed, ectopic FOXA1 repressed the expression of mesenchymal markers Vimentin (VIM) and SNAIL at the mRNA and protein levels and induced the expression of the epithelial marker CDH1 in SW480 and SW620 cells (Fig. 4e-f, Fig. S4b-c). Repression of RBM47 by siRNAs prevented the induction of CDH1 and down-regulation of VIM and SNAIL by FOXA1 (Fig. 4g-i, Fig. S4d-e). In addition, activation of ectopic FOXA1 resulted in the accumulation of CDH1 at the outer cell membrane which was prevented by siRNA-mediated down-regulation of RBM47 (Fig. 4i). Taken together, these results show that FOXA1 induces MET in CRC cells and that this effect is mediated, at least in part, by RBM47.

### FOXA1 suppresses migration and invasion of CRC cell via RBM47

**Fig. 5:**
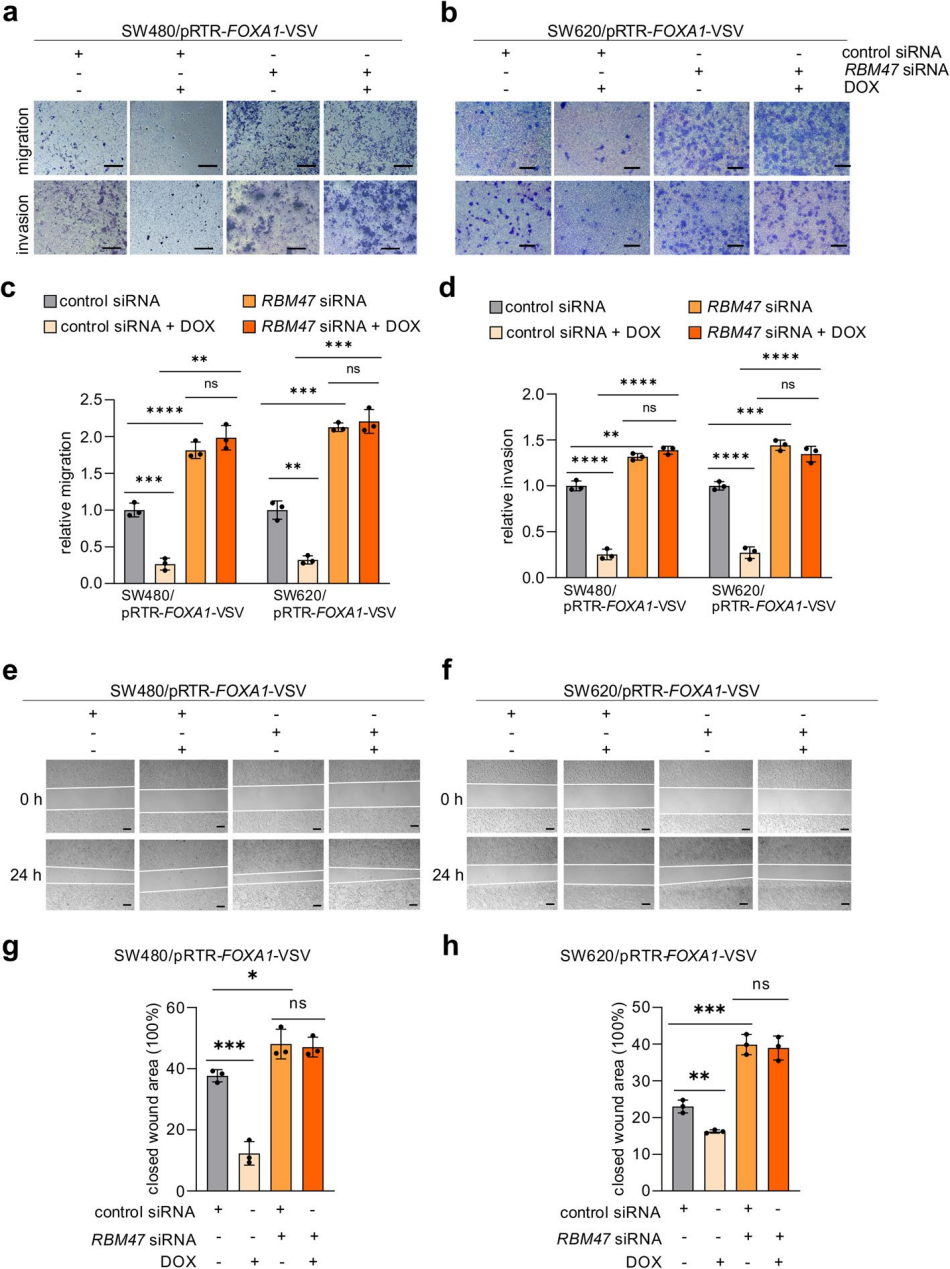
FOXA1 suppresses migration and invasion via inducing *RBM47*. **a-d** Pictures of cells **a-b** and quantification of relative migration and invasion **c-d** in Transwell chambers after ectopic expression of *FOXA1* and/or *RBM47* knock-down in SW480 and SW620 cells. Scale bars, 50 μm. **e–h** Pictures of cells **e–f** and quantification of relative migration and invasion **g-h** in wound healing assays after ectopic expression of *FOXA1* and/or *RBM47* knock-down in SW480 and SW620 cells. Mean values ± SD (*n* = 3) are provided. **p < 0.01; *** *p* < 0.001; **** *p* < 0.0001

It has been shown that EMT promotes cell migration and invasion, whereas MET suppresses these processes. Therefore, we asked whether FOXA1- and RBM47-regulated EMT/MET also affects migration and invasion of CRC cells. Ectopic expression of FOXA1 significantly attenuated cell migration and invasion when compared to the control group, as determined by transwell assays (Fig. 5a-d). In contrast, RBM47 knock-down markedly enhanced migration and invasion relative to scramble siRNA controls (Fig. 5a-d). To assess whether the repression of migration and invasion by FOXA1 relies on RBM47, SW480 and SW620 cells were transfected with *RBM47*-specific siRNAs and ectopic FOXA1 expression was induced by adding DOX. In the presence of RBM47-specific siRNAs, ectopic FOXA1 expression failed to inhibit migration and invasion (Fig. 5a-d). Also, in wound healing assays cell migration was decreased after ectopic expression of FOXA1 in cells transfected with control siRNA, but not in cells transfected with *RBM47* siRNA (Fig. 5e-h). Therefore, the suppression of migration and invasion by FOXA1 is mediated by RBM47.

### CpG methylation analysis of* RBM47*

**Fig. 6:**
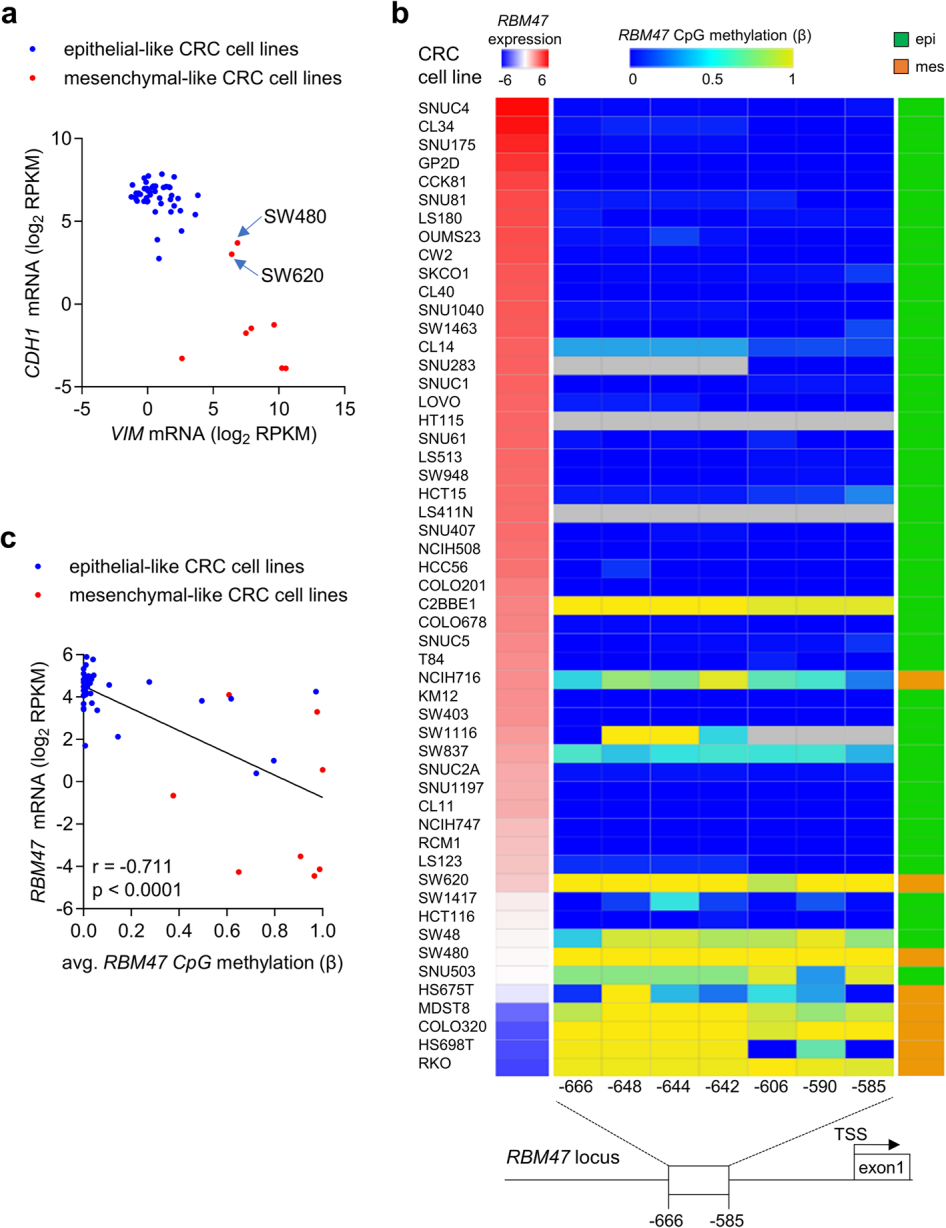
CpG methylation of *RBM47* promoter in CRC cell lines. **a** Separation of CRC cell lines into epithelial- and mesenchymal-like cells according to the expression of *CDH1* and *VIM*. **b** Association of expression and DNA methylation of *RBM47* with the epithelial/mesenchymal status of 53 human CRC cell lines (CCLE). Left red/blue heat-map: *RBM47* mRNA expression; middle blue/yellow heat-map: DNA methylation of each CpG site in the CpG island located in the *RBM47* promoter region; right green/orange heatmap: epithelial/mesenchymal status of cell lines. Bottom: the *RBM47* gene structure with indicated transcription start site (arrow) and the first exon. **c** Correlation between the *RBM47* mRNA expression and the average DNA methylation of all CpG sites in the *RBM47* CpG island in 53 human CRC cell lines. The Pearson correlation coefficient with two-tailed p-value is shown

**Fig. 7:**
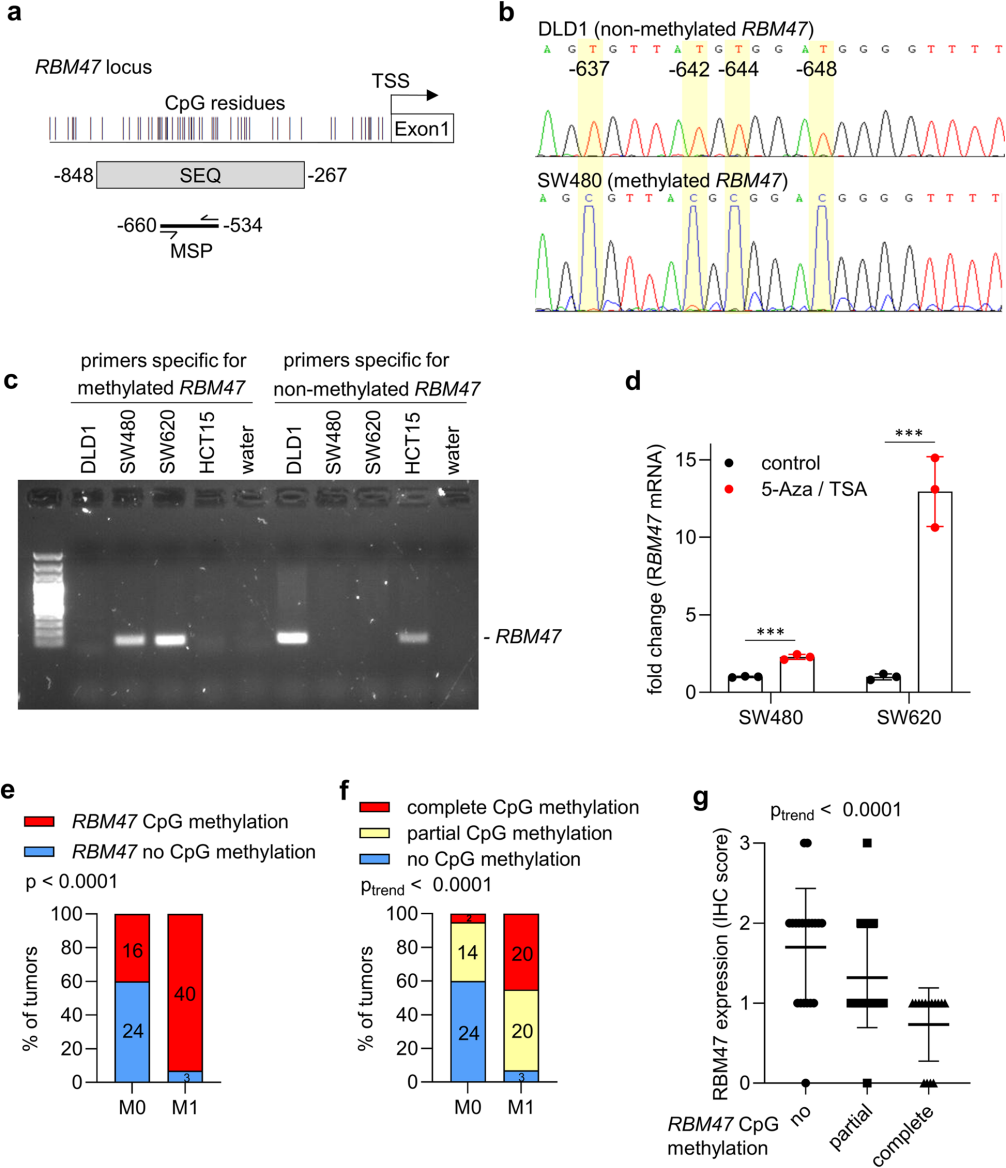
CpG methylation of *RBM47* is associated with liver metastasis in CRC patients. **a** A map of the *RBM47* promoter with regions, that were subjected to bisulfit sequencing and MSP, indicated. **b** Bisulfite-sequencing of the CpG rich region within the *RBM47* promoter in DLD1 and SW480 CRC cell lines. **c** MSP analysis of the indicated cell lines with oligonucleotides that specifically amplify the methylated and non-methylated *RBM47* promoter region. **d** *RBM47* mRNA expression in SW480 and SW620 CRC cells treated with the DNA methyltransferase inhibitor 5-aza-2’-deoxycytidine (5-Aza) for 72 h and the histone deacetylase inhibitor Trichostatin A (TSA) for the last 24 h. **e–f** MSP analysis of DNA methylation in the *RBM47* CpG island in CRC tumors from a cohort of CRC patients with (M1) or without (M0) liver metastases. **g** Correlation between DNA methylation of the *RBM47* promoter and RBM47 protein expression in CRC tumors from the M0/M1 patient cohort. ***, *p* < 0.001

Next, we aimed to determine the mechanism for the pronounced and consistent down-regulation of *RBM47* in mesenchymal-like CRC cell lines and advanced primary CRCs. A plausible mechanism for transcriptional down-regulation is the epigenetic silencing by CpG methylation [23]. Therefore, we analyzed whether the down-regulation of *RBM47* is associated with methylation of CpG residues within the vicinity of the TSS of *RBM47*. First, 53 CRC cell lines were grouped into epithelial- and mesenchymal-like cell lines, according to the expression of *CDH1* and *VIM* (data from the Cancer Cell Line Encyclopedia (CCLE)): thereby 45 cell lines were classified as epithelial-like (high expression of *CDH1* and low expression of *VIM*) and 8 cell lines as mesenchymal-like (low expression of *CDH1* and high expression of *VIM*) (Fig. 6a). Subsequently, *RBM47* expression and DNA methylation was analyzed in these cell lines (data from CCLE). Most epithelial-like cell lines displayed high *RBM47* expression and absent or low *RBM47* promoter methylation, whereas mesenchymal-like CRC-cell lines showed low *RBM47* expression and high *RBM47* promoter methylation (Fig. 6b). Accordingly, *RBM47* expression and promoter methylation in CRC cell lines showed a significant inverse correlation (Fig. 6c). To validate the differential DNA methylation at the *RBM47* promoter, bisulfite sequencing was performed and methylation-specific PCR (MSP) assays were established covering the *RBM47* promoter region that showed differential methylation in the CCLE data (Fig. 7a). Bisulfite sequencing and MSP showed that CpG-residues in this region of the *RBM47* promoter are completely non-methylated in epithelial-like CRC cell lines DLD1 and HCT15, whereas the mesenchymal-like CRC cell lines SW480 and SW620 display complete CpG methylation in this region (Fig. 7b-c). Treatment of SW480 and SW620 CRC cells with the DNA methyltransferase inhibitor 5-aza-2’-deoxycytidine and the histone deacetylase inhibitor trichostatin A (TSA) resulted in a significant re-activation of *RBM47* mRNA expression, further confirming that CpG methylation is responsible for the repression of *RBM47* expression (Fig. 7d). Next, MSP was performed to determine *RBM47* methylation in primary CRCs derived from an in-house M0/M1 cohort, which represents 43 matched pairs of CRC patients with or without liver metastases [4]. Primary CRCs from patients with liver metastases showed a significantly higher frequency and degree of *RBM47* methylation when compared those from patients without liver metastases (Fig. 7e-f). We previously analyzed the methylation of *MIR34a* and the expression of miR*-*34a microRNA, as well as c-MET, SNAIL, and β-Catenin proteins in the in-house M0/M1 cohort [24, 25]. Compared to these markers, *RBM47* methylation showed a more significant association with metastasis with a higher odds ratio, sensitivity, and specificity (Table S1). We had previously determined the protein expression of RBM47 in the same M1/M0 cohort and could show that primary tumors from patients with liver metastases display significantly lower levels of RBM47 protein expression than patients without liver metastases [4]. Correspondingly, the expression of RBM47 protein and *RBM47* promoter methylation showed a significantly negative correlation in the M0/M1 cohort (Fig. 7g). Altogether, these results show that mesenchymal-like CRC cell lines and primary CRCs that form distant metastases display silencing of the *RBM47* promoter by CpG methylation.

### *FOXA1* and *RBM47 *down-regulation during CRC progression is associated with CpG methylation of *RBM47*

**Fig. 8:**
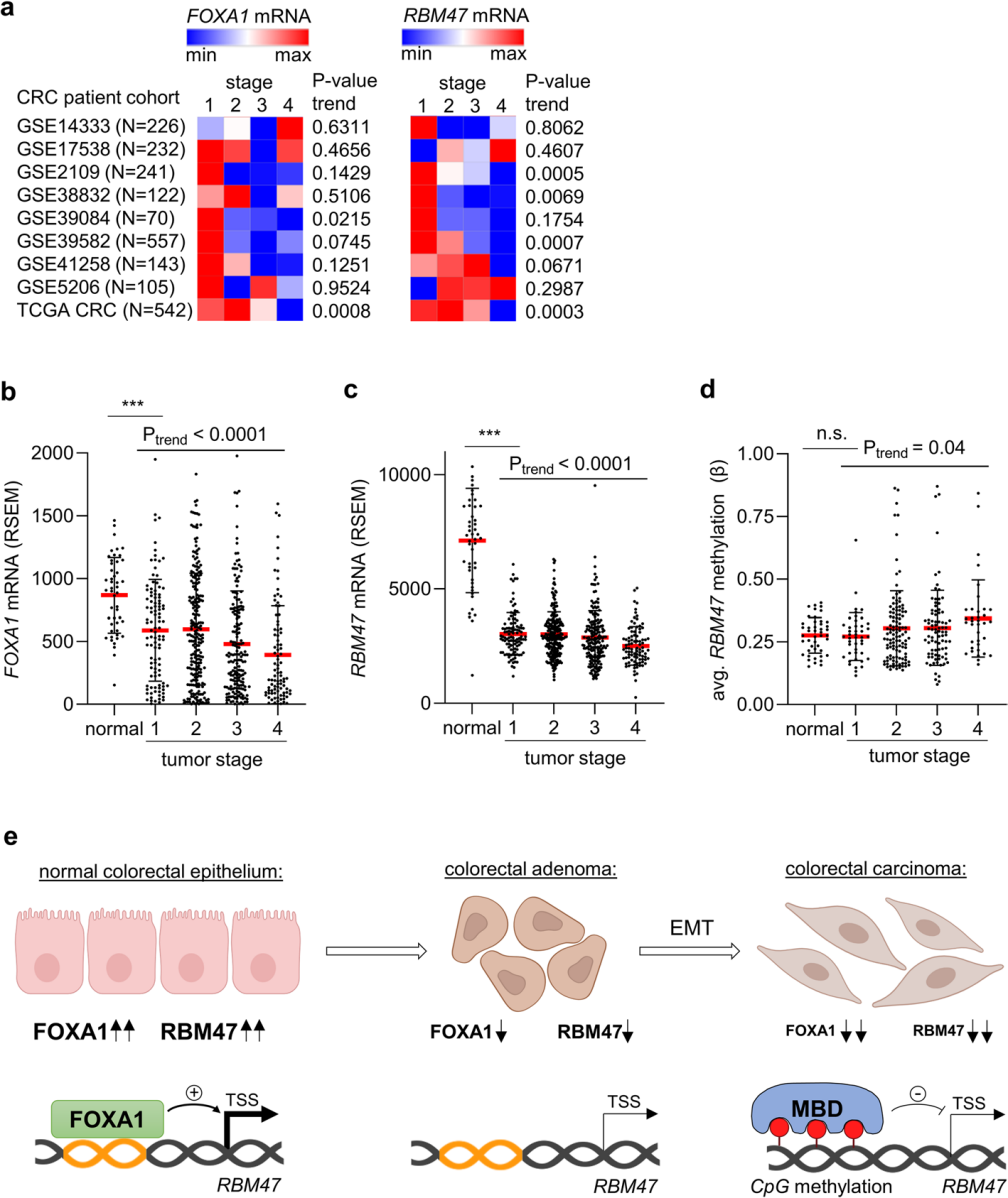
*FOXA1* and *RBM47* expression is down-regulated, while the *RBM47* promoter is hypermethylated in late stage CRCs. **a** Expression of *FOXA1* (left) and *RBM47* (right) in human CRCs belonging to indicated tumor stages in CRC patient cohorts. **b-c** Expression of *FOXA1* **b** and *RBM47* **c** in normal tissue and tumors from the TCGA COAD + READ patient cohort according to the tumor stage. **d** Methylation of the *RBM47* promoter in normal tissue and tumors from the TCGA COAD + READ CRC patient cohort according to the tumor stage. **e** A model describing the regulation of *RBM47* expression by FOXA1 and CpG methylation during CRC initiation and progression. Orange DNA: FOXA1 binding sites; Red circles: 5-methyl-cytosines; MBD: Methyl-CpG binding domain proteins; TSS: transcription start site

Since our results implied that the down-regulation of *FOXA1* and *RBM47* expression promotes tumor progression, *FOXA1* and *RBM47* expression in different stages of colorectal tumors was analyzed. Indeed, *FOXA1* and *RBM47* expression progressively decreased from stage 1 to stage 4 colorectal tumors in the majority of analyzed CRC patient cohorts (Fig. 8a). The expression of *FOXA1* and *RBM47* decreased from normal tissue to stage I tumors and further decreased in higher stage tumors (Fig. 8b and c). The tumors and normal tissue from the TCGA CRC patient cohort were also subjected to methylation profiling (Fig. 8d). Interestingly, the *RBM47* promoter methylation was similar in normal tissue and stage I tumors, but increased in higher stage tumors, suggesting the *RBM47* promoter hypermethylation does not occur during tumor initiation, but during tumor progression.

## Discussion

The results presented here show that the FOXA1–RBM47 axis is a critical regulator of MET in CRC. In normal intestinal epithelium *FOXA1* expression is high, which presumably maintains the high expression of *RBM47* in the epithelial cells. In early stage CRCs expression of FOXA1 and RBM47 is decreased. In metastatic, stage 4 CRCs an increase of epigenetic silencing of *RBM47* by CpG methylation was detected. Therefore, the following scenario appears likely (see also Fig. 8e): During the initiation of early adenomas, the expression of *FOXA1* and consequently *RBM47* is decreased. During adenoma to carcinoma progression the *RBM47* promoter becomes increasingly hypermethylated, suggesting that the initial downregulation of *RBM47*, at least in part, due to the down-regulation of FOXA1 is epigenetically fixed. In support of this model, it has been reported that FOXA1 has the ability to open condensed chromatin structures and its recruitment to enhancers and promoters is associated with DNA demethylation and induction of histone H3K4 methylation, which results in transcriptional activation [26]. On the contrary, *FOXA1* knock-down was associated with increased CpG-methylation in regions otherwise occupied by FOXA1 [27]. In addition, FOXA1 interacts with ten-eleven translocation methylcytosine dioxygenase 1 (TET1) and TET2 DNA hydroxylases [28], which oxidize methyl groups and thereby convert 5-methylcytosines into 5-hydroxymethylcytosines, 5-formylcytosines and 5-carboxylcytosines, ultimately leading to the removal of DNA methylation [29]. Therefore, the down-regulation of FOXA1 during CRC tumorigenesis would allow the CpG-methylation of the *RBM47* promoter and the repression of *RBM47* expression.

DNA methylation in cancer cells can be triggered by intrinsic and tumor microenvironmental factors. During cellular and organismal aging, DNA undergoes global hypomethylation and local hypermethylation [30]. Tumor cells with epigenetically silenced tumor suppressors are selected during progression, as they proliferate faster and out-compete other cells [31]. Furthermore, DNA methylation in CRCs can also be modulated by microenvironmental cues, such as inflammation [32] and the intestinal microbiome [33].

It has been shown that *RBM47* expression is also regulated by additional transcription factors. *RBM47* is directly repressed by the EMT-related transcription factors SNAIL and SLUG [4]. Furthermore, *RBM47* is repressed by STAT3 [4] after exposure to IL6. Consequently, the repression of *RBM47* may result from a combination of a decrease of FOXA1 expression and the activation of the *RBM47*-repressing factors, such as STAT3, during tumor progression.

It has been previously reported that FOXA1 suppresses EMT and induces MET [14, 34]. Here, we showed that the induction of MET and suppression of migration and invasion by FOXA1 is mediated by RBM47 in CRC cells. FOXA1 is highly expressed in the foregut endoderm, where its expression is induced by endodermal transcription factors, such as SOX17 [35]. FOXA proteins are required for normal development of endoderm-derived epithelia, which line the intestine and lung [12]. Mice with intestinal *Rbm47* deficiency had abnormally shaped villi in the small intestine and spontaneously developed intestinal and colonic polyps, implying a tumor-suppressive role of *Rbm47* in the intestine [7]. In addition, the majority of whole body *Rbm47*-deficient mice died before birth, while those that survived to adulthood were strikingly smaller in size than wild-type siblings [19].

The results presented here, demonstrate that RBM47 is required for FOXA1-mediated regulation of EMT/MET, migration, and invasion. It has been shown that RBM47 inhibits EMT and metastasis in non-small cell lung carcinoma by binding to and stabilizing the Axin 1 (*AXIN1*) mRNA, which suppresses WNT signaling [36]. Similarly, RBM47 also binds to and stabilizes the *DKK1* mRNA, which encodes another inhibitor of WNT signaling [6]. Thereby, RBM47 suppresses breast cancer progression and metastasis. RBM47 regulates the alternative splicing of numerous transcripts during EMT [9]. In epithelial cells RBM47 cooperates with epithelial splicing regulatory proteins (ESRPs) to promote epithelial cell-specific exon inclusion. However, in mesenchymal cells RBM47 and ESRP levels are decreased, which promotes mesenchymal cell-specific exon inclusion QKI and RBFOX2. Kim et al. showed that RBM47 regulates the alternative splicing of the tight junction protein 1 (TJP1): RBM47 promotes the formation of an alternative spliced TJP1 isoform, which is less active in the assembly of actin stress fibers and thereby suppresses cell migration [10]. Consequently, RBM47 down-regulation favors normally spliced TJP1, which enhances actin stress fiber assembly and cell migration. Whether these mechanisms are also involved in FOXA1/RBM47-mediated regulation of EMT/MET in CRC should be investigated in future studies.

The study presented here has some limitations, that should be addressed in future research. It remained unclear whether down-regulation of FOXA1 is required for the observed silencing of *RBM47* by CpG methylation and whether CpG methylation of RBM47 prevents its induction by FOXA1 during CRC progression. While this manuscript focuses on the regulation and upstream factors of RBM47, future studies should identify pathways and targets downstream of RBM47 that mediate tumor suppression by the FOXA1/RBM47 axes.

Altogether, this study establishes the FOXA1/RBM47 axis as a key component in the maintenance of epithelial cell identity, since RBM47 was identified as a required mediator of FOXA1-induced MET. Our findings further suggest a temporal order of transcriptional down-regulation of *RBM47* due to decreased FOXA1 expression and subsequent epigenetic silencing of *RBM47* by CpG methylation during CRC progression. The down-regulation of FOXA1 and RBM47 then presumably allows the conversion of epithelial-like CRC cells to more mesenchymal-like, invasive CRC cells, which ultimately result in metastases formation. Finally, our results indicate that the CpG hypermethylation of the *RBM47* promoter represents a potential biomarker for metastatic CRC.

## Materials and methods

### Cell culture, treatments and transfections

SW480, SW620, DLD1, HCT15, HT29 and CaCO-2 cell lines were cultured in McCoy`s 5 A Medium (Invitrogen) supplemented with 100 U/mL penicillin,10% fetal bovine serum (FBS, Invitrogen), and 0.1 mg/mL streptomycin at 37 °C. Doxycycline (DOX; Sigma, St Louis, MO) was used at a final concentration of 100 ng/mL. Cells were transfected with siRNAs using the Lipofectamine™ RNAiMAX (Invitrogen) in accordance with the manufacturer's guidelines. FlexiTube siRNAs (pools of four distinct siRNAs) targeting FOXA1 as well as control siRNAs were obtained from Qiagen. 5-Aza-2’-deoxycytidine (Sigma) and Trichostatin A (Sigma) were used at concentrations of 1 μM and 300 nM, respectively.

### Generation of pRTR-FOXA1-VSV pools

Stably transfected cell pools were generated as described previously [24]. PCR was used to amplify the human *FOXA1* coding region from the pLX302_FOXA1-V5 plasmid (Addgene, #70,090) and cloned into a pRTR plasmid. pRTR plasmids were transfected into cells using the Lipofectamine LTX Reagent (Invitrogen). Selection was performed with 8 μg/mL Puromycin (Sigma) for two weeks to obtain cell pools in which more than 80% of cells expressed the fluorescent marker encoded by the pRTR vector. The homogeneity of the cell pools was assessed by the treatment of cells with 100 ng/mL DOX for 48 h followed by GFP expression evaluation using flow cytometry.

### Western blot analysis

Cells were lysed in RIPA buffer (250 mM NaCl, 50 mM Tris/HCl pH 8.0, 0.1% SDS, 1% NP40, and 0.5% sodium deoxycholate) supplemented with cOmplete™ Protease Inhibitor Cocktail (Roche) and phosphatase inhibitor tablets (Roche). Cell lysates were sonicated for 5 s at 70% intensity and centrifuged at 13,000 × g for 20 min at 4 °C. Protein concentrations were determined using a BCA assay (Thermo Fisher, #23,227). A total of 30 μg protein per sample was loaded on 10% SDS-PAGE gels (Bio-Rad Mini-PROTEAN®) and transferred to PVDF membranes (Millipore Immobilon®, IPVH00010) using the Trans-Blot® Turbo™ system (Bio-Rad). Blocking of membranes was performed using 5% non-fat milk in TBST. Membranes were incubated with primary antibodies overnight at 4 °C (Table S2), followed by incubation with HRP-conjugated secondary antibodies for 1h. Chemiluminescent signals were generated with ECL Prime (Cytiva, #RPN2232) and detected using an Odyssey® FC Imager (LI-COR, 3.0 software). Antibody details are provided in Table S2.

### RNA isolation and quantitative real time polymerase chain reaction (qPCR) analysis

The High Pure RNA Isolation Kit (Roche) was used to extract total RNA from cells in accordance with the manufacturer's guidelines. The concentration and purity of RNA were analyzed with a NanoDrop™ 2000 spectrophotometer (Thermo Fisher Scientific), ensuring A260/A280 ratios of 1.8–2.0. 1 μg of total RNA was reverse transcribed to synthesize cDNA using the Verso cDNA synthesis kit (Thermo Fisher Scientific, Waltham, MA). qPCR amplification was performed using the SYBR® Green Master Mix (Applied Biosystems, Foster City, CA) on a LightCycler® 480 System (Roche). Each 15 μL PCR reaction contained 7.5 μL SYBR Green mix, 0.5 μM forward and reverse primers (sequences listed in Table S3), 1 μL cDNA template, and nuclease-free water. PCR conditions were: 95 °C for 30 s (initial denaturation), 40 cycles of 95 °C for 5 s and 60 °C for 30 s. Melt-curve analysis (65–95 °C) was used to verify primer specificity. All samples were analyzed in triplicate. The _ΔΔ_Ct method [37] was used to calculate relative mRNA expression with *GAPDH* as normalization control. The sequences of qPCR oligonucleotides are shown in Table S3.

### Immunofluorescence (IF) analysis

Cells grown on glass cover slides were fixed with 4% paraformaldehyde for 15 min and washed with PBS (3 × 5 min). After permeabilization with 0.1% Triton X-100 for 10 min and subsequent PBS washing, cells were blocked with 1% BSA for 1 h. Incubation with primary antibodies was performed overnight at 4°C in a humidified chamber. After washing with PBS, samples were incubated with Alexa Fluor-conjugated secondary antibodies (1:1000) for 1 h at room temperature in the dark. DNA was counterstained with DAPI (1 μg/mL, 5 min), and fluorescence images were acquired using a Zeiss LSM 700 confocal microscope using a 63 × objective. Information of all antibodies are provided in Table S2.

### Quantitative chromatin immunoprecipitation (qChip) analysis

qChIP analyses were performed using the Ideal ChIP-qPCR Kit (Diagenode, Cat# C01010180) in accordance with the manufacturer's guidelines. Briefly, cross-linked chromatin from 25 million cells was sonicated with a Bioruptor® Pico sonication device to obtain 200–500 bp DNA fragments. Immunoprecipitation was performed overnight at 4°C with 3 μg of FOXA1 ChIP Grade antibody (Abcam, ab170933) along with a matched isotype control. Protein-DNA complexes were captured with Diagenode optimized magnetic beads and subjected to sequential washes. Following cross-linking reversal and DNA purification using the kit columns, enriched DNA fragments were analyzed by quantitative PCR. qChIP oligonucleotide sequences are provided in Table S4.

### Transwell cell invasion and migration analysis

Cell migration and invasion were assessed using 8-μm pore Transwell® chambers (Corning, #3422). For invasion assays, inserts were covered with Matrigel® (BD Biosciences, #356,234) at a 1:8 dilution in serum-free medium and incubated for 4 h at 37 °C. Cells suspended in serum-free medium were seeded into upper chambers, while complete medium containing 15% FBS was placed in the lower chambers as a chemoattractant. After 24 h, cotton swabs were used to remove non-invaded or non-migrated cells from the upper membrane surface. Cells on the lower surface were fixed with 4% paraformaldehyde for 15 minuntes, stained with 0.1% crystal violet (Sigma, #C6158) for 20 min, and imaged using a ZEISS microscope. Three random fields per insert were quantified using ImageJ v1.53 (NIH) by threshold-based particle analysis.

### Wound healing cell migration analysis

Confluent cells in 2-well cell culture inserts (Ibidi, Martinsried, Germany, #80,241) were treated with mitomycin C (10 ng/mL) for 2 h to inhibit proliferation. After treatment, the insert was removed to generate a cell-free gap. After 0 and 24 h images of the wound area were captured using the Zeiss Axiovert Observer Z.1 microscope, AxioCam MRm camera and ZEN 3.1 software. Gap area (A) was analyzed using ImageJ and wound closure (%) was calculated as [(A_(t = 0)—A_t)/A_(t = 0)] × 100. Experiments were performed in triplicates.

### Tissue samples

A cohort of 86 colon cancer patients who underwent surgical tumor resection at the Ludwig-Maximilians University of Munich (LMU) [24, 25, 38] was utilized to analyze *RBM47* methylation (The clinicopathological characteristics are provided in Table S5). Follow-up information was obtained from the Munich tumor registry. All tumors originated from the right side of the colon. 43 patients showed synchronous liver metastases, which was confirmed by liver biopsy or imaging. 43 colon cancer patients without distant metastases at diagnosis and with a minimum disease-free survival of 5 years after primary surgery served as controls. Case and control samples were matched for T-classification (TNM 2009) and tumor grade (WHO 2000), yielding 43 matched pairs. The study was approved by the LMU Medical Faculty ethics committee and all procedures adhered to relevant guidelines and regulations.

### Isolation and bisulfite treatment of genomic DNA

Genomic DNA from cell lines was extracted using a DNeasy Blood & Tissue Kit (Qiagen). For formalin-fixed, paraffin embedded (FFPE) tissue samples, genomic DNA was isolated from 5 µm-paraffin sections following overnight digestion with 0.1 mg/mL proteinase K in 0.1% SDS (Sigma) at 58°C and extraction with phenol/chloroform at pH 8. Approximately 400 ng of DNA was subjected to sodium bisulfite conversion using an EZ DNA Methylation Kit (Zymo Research).

### Bisulfite sequencing

Bisulfite-converted genomic DNA was used as a template to amplify the *RBM47* promoter region TSS-848 – TSS-267 by PCR using the Promega GoTaq PCR mix (Promega). Following an initial denaturation 95 °C for 10 min, 38 PCR cycles were performed consisting of 95 °C for 30 s, 65 °C for 30 s, and 72 °C for 60 s, followed by an elongation at 72 °C for 10 min. PCR products were sequenced using a BigDye terminator v1.1 kit and a 3700 capillary sequencer. The sequences of oligonucleotides used for bisulfite sequencing are listed in Table S6.

### Methylation-specific PCR (MSP)

MSP was performed in a 20 μL reaction using Promega GoTaq PCR mix (Promega) and 30 ng of bisulfite-converted DNA. PCR conditions were: 95 °C for 10 min, 37 cycles of 95 °C for 30 s, 58 °C for 30 s, and 72 °C for 30 s, with a final elongation at 72 °C for 10 min. The sequences of MSP oligonucleotides are provided in Table S6.

### Bioinformatics analysis of public datasets

For the TCGA colon adenocarcinoma (COAD) and rectal adenocarcinoma (READ) cohorts clinical and RSEM normalized expression data was obtained from the MD Anderson standardized data browser (http://bioinformatics.mdanderson.org/TCGA/databrowser/) in August 2021. For additional CRC patient datasets, expression and clinical data was obtained from NCBI GEO (www.ncbi.nlm.nih.gov/geo) in August 2021. The statistics for survival analysis was calculated with a Log-rank test. For binary classification of cases into high and low expression groups, optimal cutoff values were determined using the Survminer R-package (https://CRAN.R-project.org/package=survminer). The CMS and CRIS classification for patient datasets was obtained from Guiney et al. [17] and Isella et al. [18]. The methylation (β-values) and expression (RPKM) data of CRC cell lines was downloaded from the cancer cell line encyclopedia (https://sites.broadinstitute.org/ccle/) in January 2022. The expression data from normal human tissues was obtained from GTEx (https://www.gtexportal.org/home/) in October 2022.

### Statistical analysis

Correlations were calculated using Pearson correlation coefficients and two-tailed p-values. Unpaired, two tailed Student’s t tests were used to determine the statistical differences between two groups. One-way analysis of variance (ANOVA) with the Tukey’s multiple comparison post-test were used to determine the statistical differences between more than two groups. Data is presented as the mean ± SD. All analyses were conducted using GraphPad Prism software 10.4.1. *P*-values < 0.05 were considered significant (*, *p* < 0.05; **, *p* < 0.01; ***, *p* < 0.001).

## Supplementary Information

Supplementary Material 1.

## Data Availability

TCGA colon adenocarcinoma (COAD) and rectal adenocarcinoma (READ) datasets was obtained from the MD Anderson standardized data browser (http://bioinformatics.mdanderson.org/TCGA/databrowser/). Other CRC patient datasets were downloaded from NCBI GEO (www.ncbi.nlm.nih.gov/geo). The expression and methylation data of CRC cell lines was downloaded from the cancer cell line encyclopedia (https://sites.broadinstitute.org/ccle/). The expression data from normal human tissues was downloaded from GTEx (https://www.gtexportal.org/home/).
