# Supplementary material for: Down-regulation of RBM47 due to diminished activation by forkhead box A1 (FOXA1) and silencing by CpG methylation is associated with epithelial-mesenchymal transition and metastasis of colorectal cancer
Source: Mol Biomed. 2025 Dec 3;6:133. doi: 10.1186/s43556-025-00382-4 (PMC12675864; doi:10.1186/s43556-025-00382-4)
Supplement: Supplementary file 1 — Supplementary Material 1. [file 43556_2025_382_MOESM1_ESM.pdf]

Supplementary information for:

Down-regulation of *RBM47* due to diminished activation by forkhead box A1 (FOXA1) and silencing by CpG methylation is associated with epithelial-mesenchymal transition and metastasis of colorectal cancer

Matjaz Rokavec[1*], Yuyun Du[1*], and Heiko Hermeking[1,2,3]

[1] Experimental and Molecular Pathology, Institute of Pathology, Faculty of Medicine, Ludwig-Maximilians-Universität München, Thalkirchner Strasse 36, 80337 Munich, Germany

[2] German Cancer Consortium (DKTK), Partner site Munich, 80336 Munich, Germany

[3] German Cancer Research Center (DKFZ), 69120 Heidelberg, Germany

*These authors contributed equally to this work

Correspondence: Heiko Hermeking, e-mail: heiko.hermeking@med.uni-muenchen.de

Inventory of supplementary information

- **Figure S1.** Related to Fig. 1 and 2
- **Figure S2.** Related to Fig. 2
- **Figure S3.** Related to Fig. 3
- **Figure S4.** Related to Fig. 4
- **Table S1.** Related to Fig. 7
- **Table S2.** Antibodies
- **Table S3.** Oligonucleotides used for qPCR
- **Table S4.** Oligonucleotides used for qChIP
- **Table S5.** Clinicopathological characteristics of the in-house M0/M1 cohort
- **Table S6.** Oligonucleotides used for bisulfite sequencing and methylation-specific PCR

Figure S1. Related to Fig. 1 and 2

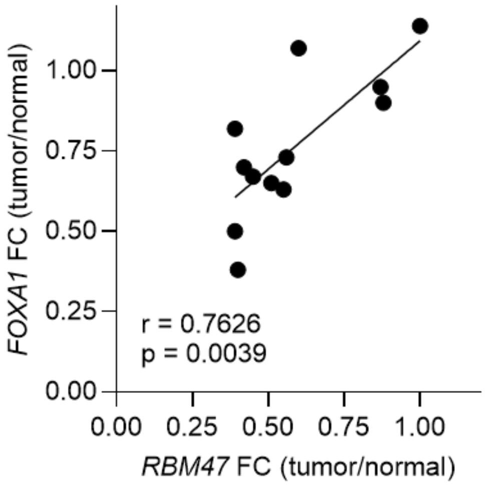

The correlation of fold changes (FC) in *RBM47* and *FOXA1* expression between tumor and normal tissues within the CRC patient cohorts analyzed in Fig. 1a and 2d. The Pearson correlation coefficient with two-tailed p-value is shown.

Figure S2. Related to Fig. 2h

Replicate 2:

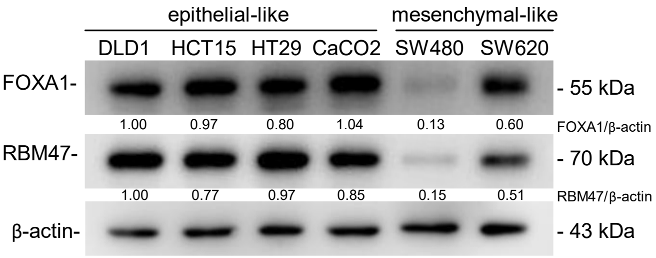

Replicate 3:

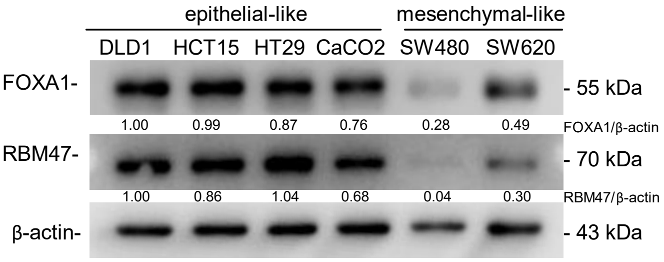

Western blot analysis of FOXA1 and RBM47 protein expression across a panel of epithelial-like and mesenchymal-like colorectal cancer cell lines. Related to Fig. 2h.

Figure S3. Related to Fig. 3

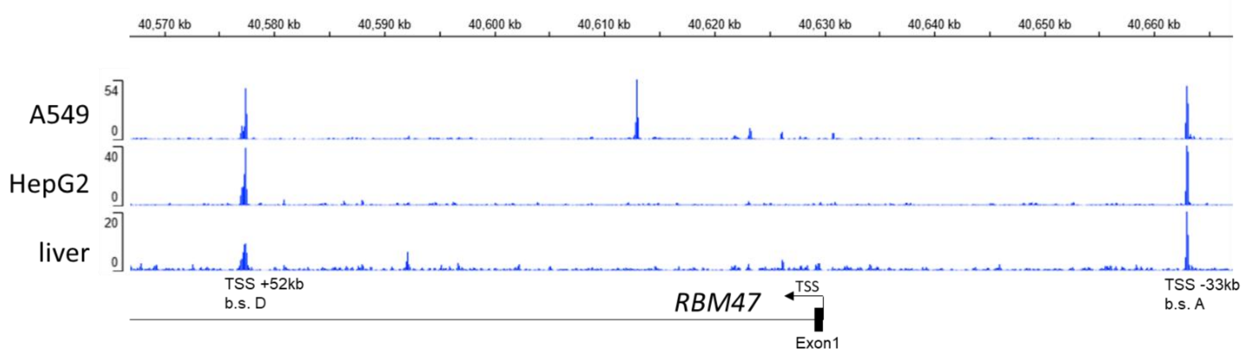

Binding of FOXA1 to the *RBM47* locus in the indicated cell lines and tissues represented by FOXA1 ChIP-seq signals. Numbers on the y-axis indicate ChIP-Seq reads. Source: The Encyclopedia of DNA Elements (ENCODE) Consortium. Visualization was done with the Integrative Genomics Viewer (IGV, Broad Institute). TSS: transcription start site. b.s.: binding site.

Figure S4. Related to Fig. 4

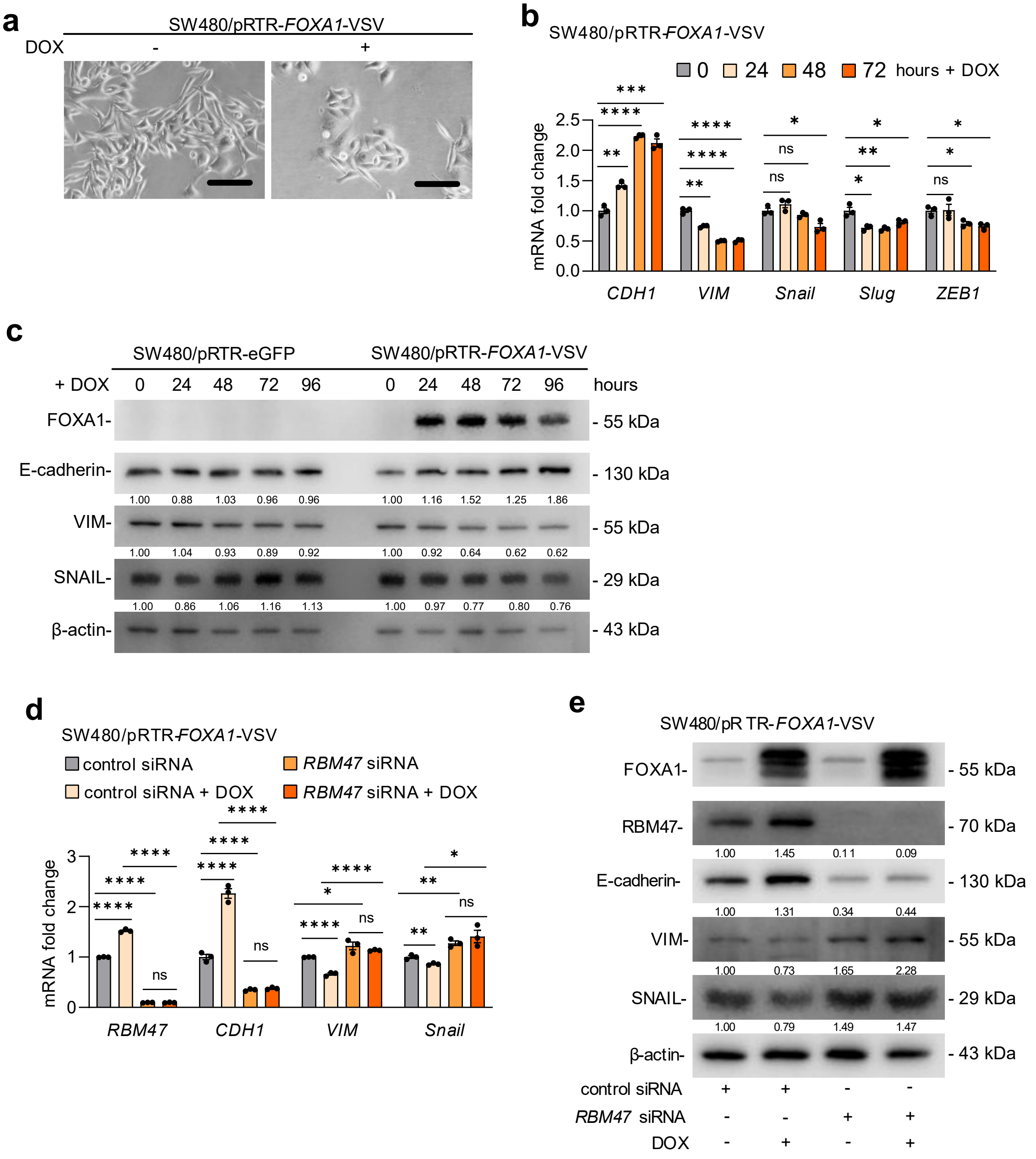

a Representative phase-contrast images of SW480 cells after ectopic FOXA1 expression by DOX treatment for 72 hours. Scale bars, 50 µm. **b-c** qPCR and Western blot analyses of FOXA1, CDH1, VIM, and SNAIL mRNA and protein expression in SW480 cells transfected with pRTR-*eGFP* or pRTR-*FOXA1* vectors, treated with or without doxycycline (DOX) for the indicated time points. **d-e** qPCR and Western blot analyses of MET/EMT markers (CDH1, VIM, SNAIL) after ectopic expression of FOXA1 and/or RBM47 knockdown in SW480 cells. Mean values ± SD (n=3) are provided. *P<0.05; **P<0.01; *** P<0.001; **** P<0.0001.

Table S1: Related to Fig. 7.

Fisher's exact test	*RBM47* methylation	*miR-34a* methylation	*miR-34a* expression	*c-Met* expression	*Snai1* expression	β-catenin
Odds Ratio	21	5.469	0.5983	3.938	1.938	2.88
p-value	<0.0001	0.0002	0.302	0.0031	0.1438	0.0299
Sensitivity	0.8889	0.7447	0.5745	0.5745	0.6596	0.766
Specificity	0.7241	0.6522	0.5532	0.7447	0.5	0.4681

Association of indicated factors with liver metastasis in CRC patients. The calculations of Odds Ration, p-value, sensitivity, and specificity for *miR-34a* CpG methylation, and *miR-34a*, *c-Met, Snai1*, and *β-catenin* expression is based on the data from Siemens et al. 2013. Clinical Cancer Research (19). 710-720.

Table S2: Antibodies

Epitope	Species	Catalog No.	Company	Assay	Dilution	Source
Primary antibodies						
RBM47	Human	ab167164	Abcam	WB	1:1000	rabbit
FOXA1	Human	ab23738	Abcam	WB	1:1000	rabbit
Vimentin	Human	# 5741	Cell Signaling Technology	WB	1:1000	rabbit
Snail	Human	# 3879	Cell Signaling Technology	WB	1:1000	rabbit
E-cadherin	Human	# 33-4000	Invitrogen	WB	1:1000	mouse
				IF	1:1000	
β-actin	Human	# A2066	Sigma-Aldrich	WB	1:1000	rabbit
Secondary antibodies						
Anti-mouse HRP	N.A.	# W4021	Promega	WB	1:10000	goat
Anti-rabbit HRP	N.A.	# A0545	Sigma-Aldrich	WB	1:10000	goat
Alexa Fluor Plus 555	N.A.	# A32727	Thermo Fisher	IF	1:1000	goat

Table S3: Oligonucleotides used for qPCR

mRNA	forward (5'-3')	reverse (5'-3')
GAPDH	TGTTGCCATCAATGACCCCTT	CTCCACGACGTACTCAGCG
β-actin	TGACATTAAGGAGAAGCTGTGCTAC	GAGTTGAAGGTAGTTTCGTGGATG
RBM47	CCTCATGATGGACTTTGACG	GCGGATCTCGTAGTTGTTGAG
FOXA1	GCAATACTCGCCTTACGGCT	TACACACCTTGGTAGTACGCC
CDH1	CCCGGGACAACGTTTATTAC	GCTGGCTCAAGTCAAAGTCC
VIM	TACAGGAAGCTGCTGGAAGG	ACCAGAGGGAGTGAATCCAG
SNAIL	GCACATCCGAAGCCACAC	GGAGAAGGTCCGAGCACAC
Slug	GGGGAGAAGCCTTTTTCTTG	TCCTCATGTTTGTGCAGGAG
ZEB1	TCAAAAGGAAGTCAATGGACAA	GTGCAGGAGGGACCTCTTTA

Table S4: Oligonucleotides used for qChIP

gene	forward (5'-3')	reverse (5'-3')
RBM47 (site A)	CTCTTCTCTCTGAGAGCAATAGGAGTT	GGCCATATTTGAACAGTGGAAAT
RBM47 (site B)	GGGATTTCACTATGTTGGTCAGG	GAATTGACTCACCGATAGTTTTAGGA
RBM47 (site C)	ATGAAGACACTAAAACTCAAAGCGAT	GCATTTTAGTTTTCTAGACCCTTGTCT
RBM47 (site D)	AAACAGAATCTAACCAGGTACCCATT	GCTTCAGCTCTGTGCATGCTG
TFF1	CACCCCGTGAGCCACTGT	CTGCAGAAGTGATTCATAGTGAGAGAT
16q22	CTACTCACTTATCCATCCAGGCTAC	ATTTCACACACTCAGACATCACAG

Table S5: Clinicopathological characteristics of the in-house M0/M1 cohort

Characteristics	Total
All patients	86 (100)
Age (y, Median 68)	
< 68	41 (48)
≥ 68	45 (52)
Gender	
Male	41 (48)
Female	45 (52)
Tumor size (UICC)	
T2	8 (9)
T3	63 (73)
T4	15 (18)
Nodal status	
N0	38 (44)
N+	48 (56)
Metastasis (Liver)	
M0	43 (50)
M1	43 (50)
Tumor grade (WHO)	
Low	30 (35)
High	56 (65)

Percent values are given in parentheses

Table S6: Oligonucleotides used for bisulfite sequencing and methylation-specific PCR

Primer	forward (5'-3')	reverse (5'-3')
RBM47 bisulfite seq	TGGTTTTGATGGTAGAGATTGGG	AAATTCAAACCAACTCCCCAAAA
RBM47 MSP methylated	TTTTGCGATTTTCGCGCGTTCG	CAAAACCCCGTCCGCGTAACG
RBM47 MSP non-methylated	TGATTTTTGTGATTTTTGTGTGTTTG	AATCAAAACCCCATCCACATAACA
